# Bioengineering Beige Adipose Tissue Therapeutics

**DOI:** 10.3389/fendo.2015.00164

**Published:** 2015-10-20

**Authors:** Kevin M. Tharp, Andreas Stahl

**Affiliations:** ^1^Program in Metabolic Biology, Department of Nutritional Science and Toxicology, University of California Berkeley, Berkeley, CA, USA; ^2^Department of Bioengineering, University of California Berkeley, Berkeley, CA, USA

**Keywords:** beige, adipose, brown fat, brown adipose, obesity, diabetes mellitus, bioengineering, hydrogel

## Abstract

Unlocking the therapeutic potential of brown/beige adipose tissue requires technological advancements that enable the controlled expansion of this uniquely thermogenic tissue. Transplantation of brown fat in small animal model systems has confirmed the expectation that brown fat expansion could possibly provide a novel therapeutic to combat obesity and related disorders. Expansion and/or stimulation of uncoupling protein-1 (UCP1)-positive adipose tissues have repeatedly demonstrated physiologically beneficial reductions in circulating glucose and lipids. The recent discovery that brown adipose tissue (BAT)-derived secreted factors positively alter whole body metabolism further expands potential benefits of brown or beige/brite adipose expansion. Unfortunately, there are no sources of transplantable BATs for human therapeutic purposes at this time. Recent developments in bioengineering, including novel hyaluronic acid-based hydrogels, have enabled non-immunogenic, functional tissue allografts that can be used to generate large quantities of UCP1-positive adipose tissue. These sophisticated tissue-engineering systems have provided the methodology to develop metabolically active brown or beige/brite adipose tissue implants with the potential to be used as a metabolic therapy. Unlike the pharmacological browning of white adipose depots, implantation of bioengineered UCP1-positive adipose tissues offers a spatially controlled therapeutic. Moving forward, new insights into the mechanisms by which extracellular cues govern stem-cell differentiation and progenitor cell recruitment may enable cell-free matrix implant approaches, which generate a niche sufficient to recruit white adipose tissue-derived stem cells and support their differentiation into functional beige/brite adipose tissues. This review summarizes clinically relevant discoveries in tissue-engineering and biology leading toward the recent development of biomaterial supported beige adipose tissue implants and their potential for the metabolic therapies.

## Introduction

Proposing brown adipose tissue (BAT) expansion as a therapeutic treatment for obesity and obesity-related disorders has recently gained significant traction (1, 2). BAT, as well as beige adipocytes (3, 4), has high metabolic capacity due to high mitochondrial content and expression of uncoupling protein-1 (UCP1) (5), a long-chain fatty acid anion/proton-symporter (6), found in the inner mitochondrial membrane. UCP1 decouples the action of ATP-synthase and dissipates the proton gradient produced by the electron transport chain, thus generating heat. BAT mass inversely correlates with body mass index (BMI), which supports the notion that BAT may regulate overall bodyweight and metabolic health (7). The benefits of expanded UCP1-expressing adipose may not be limited to their metabolic characteristics, as brown adipose has been shown to possess a potent secretome (8). Notably, implanted BAT has been shown to produce secretions of IGF-1, which was reported to enable the insulin-independent reversal of type-1 diabetes (9). BAT also appears to exert significant effects on the lipid metabolism in the liver (10) and bone mineral density (11, 12). Overall, the generation of functional beige or brown adipocytes for therapeutic purposes appears to hold significant merit for a number of possible treatments.

The current strategies to clinically deploy BAT fall into two main categories: pharmaceutical or genetic interventions to induce endogenous BAT/beige differentiation pathways, and the *ex vivo* generation of autologous cell/tissue transplants (13–17). Current gene-therapy approaches still have challenges to overcome before they are applied as anti-obesity therapeutics (18), but have proven to be significant instruments for investigating BAT biology (16, 19). Pharmacological activation of the pathways that drive a white adipose tissue (WAT) to beige/brite transition, a process known as “browning”, offer little control over the location and temporal extent of the effects. Transplantation of autologous BAT in small animal models has shown clear metabolic enhancements (20–22) but approaches are unlikely to be suitable for human therapies since there are few sources of transplantable mature human BAT and immune-rejection would need to be overcome. To successfully harness the therapeutic potential of BAT, a readily available source of transplantable cells with the potential to robustly generate UCP1-positive (UCP1+) adipocytes must first be identified or created. Second, the basis to form a functional, non-immunogenic, highly localized, and metabolically active brown/beige tissue must be defined.

## Tissues for Tissue-Based Therapies

The use of small molecules or growth factors to induce the browning of WAT is a promising area of research, but systemic dosing has the potential to produce off target effects with undesirable consequences. One notable browning agent, rosiglitazone, was widely used to treat diabetes but has disconcerting side effects that included heart failure (23). Browning factors (24–39) generally target conserved signaling pathways, making specificity a concern. By using biomaterials to localize and control release kinetics of browning factors to desired anatomical locations, an acceptable level of specificity may be achieved (40–46). When increasing the number of brown adipocytes, other supporting cell types are required for producing a functional tissue. For BAT, this includes interactions between the nervous, vascular, and immune systems, with UCP1+ adipocytes. Efforts to understand how brown or beige fat responds to cold temperature have uncovered evidence that classical brown fat relies on the sympathetic nervous system (47–49) and immune system (50, 51) to initiate and maintain the thermogenic response. While immune (33, 34) and nervous systems (52) also play a major role in beige fat development and activation, beige adipocytes have the innate ability to sense temperature and independently respond by either differentiating into UCP1+ adipose and/or by inducing uncoupled respiration (53). Thus, classical brown, but not beige, tissue therapeutics will likely require sympathetic innervation for thermogenic function and persistence. The vascular system enables access to metabolic substrates, as well as the oxygen required for the metabolic activity of brown/beige fat (10). Additionally, vascular networks circulate activating or browning signals (25, 30, 35, 54–56). In addition to innervation and vascularization, tissue-based therapies facilitate important cell–cell and cell–extracellular matrix (ECM) interactions that provide function altering chemical and physical inputs (57–62).

Natural ECM is comprised of collagens, elastins, fibronectin, laminins, proteoglycans, and glycosaminoglycans (63). The ECM is a highly organized network of physical signals that dynamically interact with the cells it supports. The topology and composition of the ECM is heavily remodeled, especially during differentiation. Remodeling of the ECM is a balance of specific degradation by matrix metalloproteinases (MMPs), new matrix component deposition, unmasking of cryptic binding sites in response to cleavage or tension, crosslinking or bonding of ECM components, and inside-out signaling from adhesion receptors on cellular surfaces. Growth factor signaling is regulated through the ECM by controlling their capture or exclusion, rate of delivery to the cell surface receptor, and molecular presentation (64). The ECM establishes a biological framework that provides physical support to cells, but also regulates signaling through adhesion receptors and alters endocrine, paracrine, autocrine, and juxtacrine signals (62). To date, many groups have remade “synthetic” tissues by decellularizing the desired tissue, then reseeding the remaining native ECM with cell populations (65, 66). However, given the inaccessibility of mature BAT, this approach will be difficult to translate to artificial UCP1+ tissues.

Tissue architecture has been known to exert strong effects on cell behavior, and *in vitro* hydrogels, meant to mimic natural ECMs, appear to be a successful option to improve cell culture models (67). These 3-dimensional (3D) hydrogels, which are water swollen polymer networks, have been utilized to enhance hepatocyte (68) and pancreatic islet function (69). Not only does 3D-organization affect the function and viability of cells (70), but it mediates the differentiation of many cell types (71), especially adipocytes. Adipogenesis is highly dependent on cytoskeletal rearrangements where cell shape is altered to accommodate the intracellular accumulation and organization of lipids. This was discovered when preadipocytes could not differentiate when cultured on a surface of fibronectin (72); this phenotype was rescued by disrupting the cytoskeletal response to the supraphysiological abundance of fibronectin. Laminin also has been found to play a role in WAT expansion (73). Another adipose ECM component, Collagen VI, was found to be an essential microenvironmental signal for adipocytes to regulate the amount of TAG they accumulate (74). When Collagen VI is removed from the adipose ECM, adipocytes develop a hypertrophic state in which the adipocytes are capable of sequestering significantly more lipids than normal. Specific ECM degradation is also a critical feature of WAT development (75). These phenomenal findings support the notion that the adipose ECM is an integral signal for adipocyte behavior and overall condition and function of the organ. When working with *in vitro* models of white adipocytes, it is also evident that the prototypical unilocular lipid droplets observed *in vivo* are not present unless cultured in 3D-matrices (76, 77).

Comparatively little is known about the ECMs of WAT, BAT, and the changes in adipose-matrix during browning. As this area of research is explored further, ECM-derived signals will hopefully be identified to build our understanding of adipocyte-ECM interactions and provide applicable systems to induce the formation of UCP1+ adipocytes. Biomaterials have utilized the incorporation of whole ECM components, such as laminin (78) or collagen (79), to improve their biocompatibility and effectiveness as cell scaffolds. Whether or not specific mixtures of ECM components can be combined with preadipocytes to form functional brown fat has yet to be determined, but this approach has already been applied to WAT (66, 80, 81). ECM molecules could theoretically be derived from the same tissues used to isolate the primary precursor cell populations for the generation of an adipose allograft (66) but thermogenic fat may have a distinct structural niche capable of engaging specific integrin (82) and syndecan (83) populations. Integrin expression is known to be highly dynamic during adipogenisis (84) and the beta-1 integrin has been extensively used to purify preadipocytes (85). Therefore, targeting integrin signaling appears to be a logical method to alter adipogenesis and it is likely that UCP1+ tissue function is dependent on specific 3D interactions and organization. This has now been demonstrated via integrin-ligands, in the form of matrix-derived peptide sequences and secreted molecules, on both UCP1-expression and lipid accumulation (86, 87).

## Materials to Build Tissues

**Figure 1:**
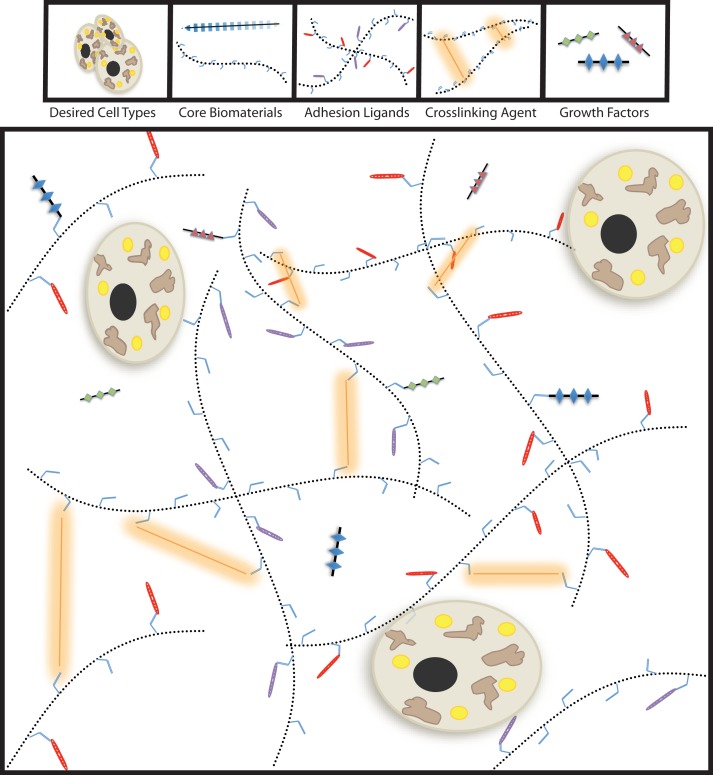
**Modular tissue-engineering**. Here, we visually depict the five bioengineering variables discussed throughout this review. By tailoring the properties biochemical and physical properties of the biomaterial, synthetic tissues for therapeutic purposes can be successfully generated.

Decellularized tissue-based tissue-engineering is not the only method to produce synthetic tissues (81, 88, 89). Polymeric biomaterials with bioactive modifications have been employed to generate many tissue types (61, 90–92). Tissue-engineering is a sophisticated endeavor that requires significant tuning, therefore modular approaches should be considered for the construction of functional synthetic tissues. Specifically, the physical properties, degradation kinetics, and biologically interactive components should be able to be altered independently. Bioengineered tissues can be generated through the selection of a core polymer, a crosslinking system, bioactive modifications, and the incorporated cell populations (Figure 1). The core material of the biomaterial determines the method of deployment (injectable-vs.-implantable), macrostructure, and the behavior of the cellular payload.

While many biocompatible materials have been proposed to engineer beige/brown-adipose tissue (93), HyA-based hydrogels show particular promise for the engineering of adipose tissue for therapeutic purposes (94, 95) and is the only material that has been successfully used to establish brown/beige fat implants *in vivo* (86). HyA is a naturally occurring glycosaminoglycan, consisting of β-1,4-d-glucuronic acid– β-1,3-*N*-acetyl-d-glucosamine, that is highly tractable for tissue-engineering for biomedical purposes (94, 95). Endogenous HyA is synthesized by hyaluronic acid synthase and extruded into the extracellular microenvironment, where it functions as an essential component of the native ECM and interacts with cell surface receptors such as CD44 or RHAMM (96). HyA is highly variable in length, spanning lengths up to 10 μM and ranging from 100 kDa to 8 mDa. Hyaluronic acid plays a pivotal role in ECM organization through its interactions with the other major components of the ECM (63).

HyA scaffolds enhance survival of autologous adipose stem-cell implants (97–100) and possibly promote adipose expansion (101). Not only are HyA-based hydrogels naturally occurring, biocompatible, modifiable, injectable, biodegradable, non-immunogenic, and anti-thrombogenic (94, 102), but also HyA has already been FDA approved for a number of clinical applications such as correction of facial lipoatrophy, wrinkle and scar removal, amelioration of osteoarthritic joint pain, dietary supplementation, ulcers, and cataract surgery. HyA has also showed clinical success for the temporary esthetic augmentation of lips, breasts, and buttocks (103), and achieved impressive results as a replacement of traditional dressings of epidermal burns and lesions (104). Importantly, utilizing HyA avoids fibrotic encapsulation of implants, a problem that has plagued the early literature of bioengineering (105). In general, avoiding encapsulation requires the use of non-immunogenic materials and cells, biodegradable materials, nearly anisotropic physical properties to the surrounding tissues, and possibly growth factors to induce recruitment of host-derived cells into the implant. HyA has been shown to have significant effects on tissue remodeling and cell signaling, and is naturally degraded by hyaluronidase or oxidizing agents (106).

## Cells for Metabolic Therapeutics

Generation of bioengineered-BAT will rely on an easily accessible and ample source of progenitor cells. A key discovery in this regard came from the observation that UCP1+ adipocytes can be generated by certain WAT depots (107). These distinct adipocytes are described as beige/brite, and they express unique surface markers (3). While genetic factors play a major role in the ability to generate beige adipocytes (108, 109), the expansion of beige adipose mass has been linked to improved metabolic health. Therefore, the isolation of adipose-derived multipotent stem cells (MSCs) from undesirable WAT depots and subsequent reintroduction as autologous BAT shows therapeutic promise. One of the most abundant sources of preadipocytes is WAT, and the implantation of WAT-derived stem cells is an FDA accepted procedure (110).

The most common source of WAT-derived stem cells is the stromal vascular fraction (SVF), which contains T cells, B cells, mast cells, adipose tissue macrophages, and MSCs such as preadipocytes and endothelial progenitor cells. This cellular fraction can be further purified to enrich preadipocytes by selecting for cell surface markers such as Pref-1+, Lin−, CD29+, CD34+, Sca-1+, CD24+, CD45−, Mac1−, PDGFRα+ (15, 17, 111). Additionally, preadipocyte sorting can be used to enrich for populations that are known to readily transdifferentiate into beige/brite fat such as CD137, TMEM26, ASC-1, PAT2, and P2RX5 (112). Alternatively, as stem-cell therapies become more accepted, induced pluripotent cells might be a suitable option since iPSC-derived brown adipocytes have also been generated and transplants of these cells show promising metabolic effects (16, 113).

While a purified population of preadipocytes or adipocytes may provide more conclusive insights for biological experiments, it may not be optimal for building a functional tissue. As previously mentioned, the immune system and vascular systems are essential for supporting the function and formation of beige adipose tissue and other SVF components could contribute to these tissue types. For example, macrophages and T cells have been shown to be an important part of beige/brown-adipose function and development (33, 51, 114) and interactions between the adipocytes and the vascular niche may also be important for browning (115–118) particularly through cell–cell interactions, cytokines, and growth factors such as Il-33 (35) or VEGF-A (119). Thus, the use of multiple purified cell population or utilization of non-purified-SVF, as recently demonstrated for the generation of bioengineered-BAT (86), may offer distinct advantages.

## Biomaterial Optimization

Degradation and remodeling ability of the synthetic ECM is just as important as the initial structure itself. If the synthetic tissues are not biodegradable through mechanisms that cells naturally use for movement and reorganization, integrating with the host will be jeopardized. Specifically, to facilitate effective remodeling and reorganization of the tissue by the immune and vascular systems, biodegradable and biologically interactive biomaterials should be utilized. The simplest way to imbue a biomaterial with biodegradability is to use MMP-sensitive crosslinking agents. Most of the available core materials can be easily modified to accommodate the current MMP-sensitive crosslinkers, which are short peptides containing an MMP-specific cleavage site (61, 120–122). Most of these core materials will be modified to facilitate efficient crosslinking by spontaneous aqueous phase reactions, such as the Michael addition where a thiol and acrylate form a thio-ether bond (92, 123). These types of biodegradable crosslinkers have been shown to be essential for the recruitment of host cells for the successful *in vivo* integration of biomaterial implants (122, 124).

Additionally, the elastic modulus of biomaterials has been shown to be highly instructive for the differentiation of MSCs into adipocytes (125–127). This mechanotransductive control of differentiation can be accomplished without applying direct physical forces to cells. By presenting a cell with an adhesion-promoting environment, matrix-associated adhesions form and produce an intrinsic mechanotransductive signal for the cell, as well as adjacent cells in the microenvironment. Therefore, a soft biomaterial optimized with specific adhesion-promoting ligands may be capable of inducing the same mechanotransductive signals as a much stiffer material. Numerous biological processes are affected by mechanical signals; notably, nuclear envelope plasticity and permeability (128, 129), splicing (130), and signal transduction (131). The prominent browning factor, BMP7, is known to alter cytoskeletal dynamics in adipocytes and other cell types, which supports the notion that physical cues may be important for bioengineered-BAT (32, 132–134). Interestingly, our group found that the storage modulus of WAT seems to differ from that of BAT (WAT ~ 3 kPa, BAT ~ 4 kPa). How important this difference in modulus is for brown fat development and function remains to be explored more systematically.

Bioactive modifications, such as integrin-binding domains conjugated to hydrogels, have become commonplace to enhance the bioactivity of biomaterials (61, 90, 91) and HyA-hydrogels augment integrin signaling (135). These materials provide some degree of ECM-mimicry without replicating the entire complexity of the native ECM. For example, alginate conjugated with RGD-containing ligands is supportive to cardiomyocytes and also promotes adipogenisis (136, 137). However, alginate’s effects on adipogenisis may be due to the rounded morphology, shown to be strongly instructive to adipogenesis (72), cells undergo when encapsulated in alginate-based materials (138). Other peptide-modified hydrogels promote the formation of bone via collagen mimetic peptides (139). ADMSC-spheroids entrapped in Poly(ethylene glycol)-hydrogels have been proposed to form beige adipocytes *in vitro* (140) and have been modified to drive MSCs toward adipogenic or osteogenic fates (141). Interestingly, Vaicik et al. find UCP1-expression highest when the storage modulus of the hydrogel is BAT-equivalent.

Recent work by our laboratory to develop bioengineered-BAT with HyA-based biomaterials, differentiation promoting adhesion ligands, MMP-sensitive crosslinkers, and ADMSCs has produced a synthetic beige adipose tissue. The discovery of these bioactive modifications came from an effort to screen for ligands that preferentially promoted the attachment of UCP1+ adipocytes. Incidentally, it was discovered that some of these bioactive components, derived from laminin, directly enhanced UCP1-expression (86). The identified bioactive peptides were then conjugated to a HyA core material to promote browning. The assembly of the optimized hydrogel with adipocytes was termed beige adipose tissue-matrix-assisted cellular transplant (BAT-MACT), and was shown to successfully scaffold beige adipocytes for therapeutic purposes in animal models. What is particularly exciting about these HyA-hydrogels and MMP-sensitive crosslinkers is that they are injectable, making this approach as non-invasive as possible. Due to HyA’s viscoelastic properties, it can be easily mixed with the desired cell populations and just before implantation, mixed with the crosslinker. This allows for a short period of time where the cell-laden hydrogel is still a liquid and can be injected to the desired anatomical location with subsequent crosslinking to solidify a functional organoid.

**Figure 2:**
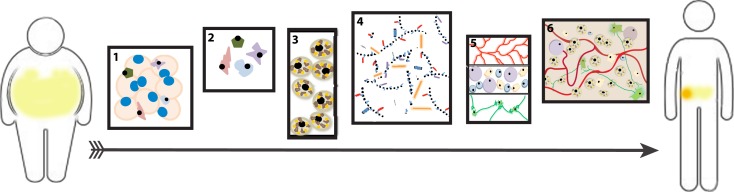
**Beige adipose tissue-matrix-assisted cellular transplant (BAT-MACT)**. The strategy used to expand beige adipose tissue for therapeutic purposes can be deconstructed into six stages: (1) Isolation of white adipose tissue (WAT) from patient. (2) Purification of stromal vascular fraction from the explanted WAT. (3) *In vitro* differentiation of SVF-derived preadipocytes toward the beige fat phenotype. (4) Suspension of beige adipocytes (SVF) into optimized hydrogel for implantation. (5) Post implantation, the hydrogel is biocompatible and degradable so that it is permissive to integrate the recipient’s vascular, immune, and nervous systems. (6) The established beige adipose tissue functions as a metabolically active organ. If a sufficient increase of metabolic rate is achieved via BAT-MACT implantation, weight loss will occur.

The BAT-MACT implant system (Figure 2) has shown that implanted beige fat can have a nearly immediate effect on glucose homeostasis that persists during the duration of the implant’s lifespan. Implanted beige adipose, similar to brown adipose, responds to cold stimulus by increasing lipid uptake an oxidation. The expansion of beige fat attenuates weight gain on a high-fat diet and induces a thermo-responsive metabolic augmentation to the recipient (86). Being that adipocytes derived from WAT sources are capable of sensing temperature independent neuronal signaling, it is not surprising that an implant of beige fat behaves this way. However, implanted classical brown adipose may not necessarily function in this manner, as the organ relies heavily on the sympathetic neuronal stimulation to induce the thermogenic program.

## Future Directions

The first iteration of BAT-MACTs had an *in vivo* lifespan of approximately 3–4 weeks (86), which allows for well-controlled spatio-temporal applications but may be too short for single-application-based metabolic interventions. Further effort is needed to understand what would promote the maintenance of such a metabolically active depot for longer periods of time and if a microenvironment capable of promoting beige adipose self-renewal could be created (71). The inclusion of modifications that enhance beige adipogenesis or metabolic activity, such as sequestered growth factors, small molecules, or mixed materials will certainly be pursued. Incorporating metabolic activators of beige adipocytes into the BAT-MACT system may be an essential step toward promoting weight loss and metabolic improvement in the face of thermoneutrality. At this stage, by using immobilized synthetic physical cues, the biomaterial component has a significantly lower risk of off target effects relative to a similarly functioning implant generated with secreted factors temporarily sequestered within the matrix. At its extreme, the implanted matrix could be endowed with sufficient biochemical and biophysical clues for the recruitment and directed differentiation of stem cells to allow for cell-free implants that are sufficient to establish beige adipose tissue. Such an acellular implant would be attractive as it could avoid many cost and health concerns associated with cellular implants.

Overall, extension of tissue-engineering principles to brown fat biology holds the promise of furthering both our mechanistic understanding of the factors required for brown/beige fat function and development, the rapid testing of hypothesis regarding the physiological impact of brown/beige tissue expansion, and ultimately novel treatment options for metabolic disorders.

## Conflict of Interest Statement

The authors declare that the research was conducted in the absence of any commercial or financial relationships that could be construed as a potential conflict of interest.
